# Two cases of left recurrent laryngeal nerve paralysis after right superior mediastinal node dissection

**DOI:** 10.1186/s40792-021-01236-1

**Published:** 2021-06-28

**Authors:** Yasuyuki Nakamura, Yuma Shindo, Wataru Arai, Kodai Tsuruta, Ryunosuke Maki, Masahiro Miyajima, Atsushi Watanabe

**Affiliations:** Department of Thoracic Surgery, Sapporo Medical University School of Medicine, South-1, West-16, Chuo-ku, Sapporo, 060-8543 Japan

**Keywords:** Lung cancer, Thoracoscopic surgery, Node dissection, Recurrent laryngeal nerve paralysis

## Abstract

**Background:**

Ipsilateral recurrent laryngeal nerve paralysis is one of the rare complications during the superior mediastinal node dissection for lung cancer. However, very few reports of contralateral recurrent laryngeal nerve paralysis during the procedure are available.

**Case presentation:**

Two women aged 74 and 80 years developed hoarseness after undergoing right upper lobectomy and right superior mediastinal node dissection for primary lung cancer. Postoperative laryngoscopy in the two patients confirmed left vocal cord paralysis.

**Conclusion:**

Node dissection is performed in the standard procedure for right upper lobe lung cancer. At this time, care must be taken not to cause damage not only to the recurrent laryngeal nerve on the ipsilateral side but also to the recurrent laryngeal nerve on the contralateral side.

## Background

Vocal cord paralysis may occur as a complication during lung cancer surgery due to recurrent laryngeal nerve injury during the superior mediastinal node dissection. Lymphadenectomy during surgery for right upper lobe lung cancer involves superior mediastinal node dissection, but very few reports of the left recurrent laryngeal nerve paralysis during surgery for right-lung cancer are available. This paper reports two cases of left recurrent laryngeal nerve paralysis after surgery for the right upper lobe lung cancer.

## Case presentation

### Case 1

**Fig. 1:**
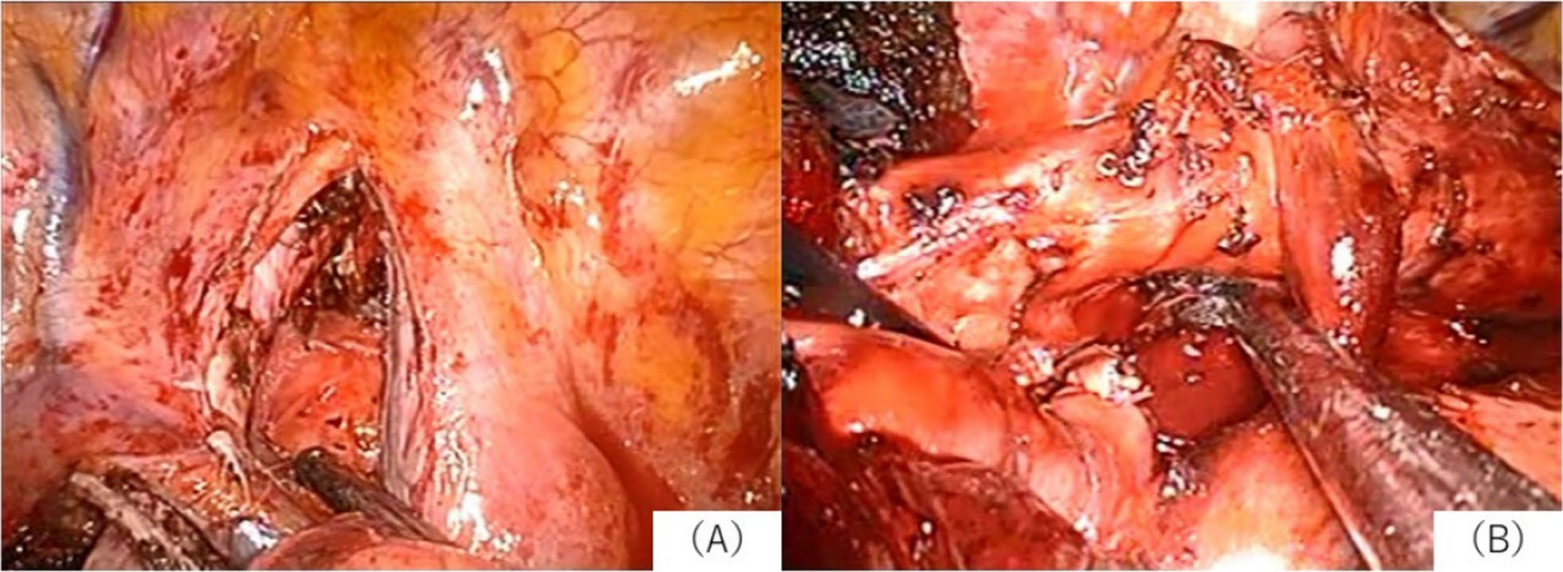
Case 1: this figure shows the findings after the upper longitudinal node dissection. **A** is after the dissection of # 2R, and **B** is after the dissection of # 4R

A 74-year-old woman was admitted to our hospital with suspected primary lung cancer in the right upper lobe clinical T1bN0M0, stage IA2. Right upper lobectomy and superior mediastinal node dissection were performed under general anesthesia with selective lung ventilation using a double-lumen tube. Nodes in the right upper paratracheal nodal station (# 2R) and right lower paratracheal nodal station (# 4R) were dissected. Nodes in # 2R and # 4R showed no swelling, but were removed contralaterally along the anterior surface of the trachea (Fig. 1). The operation was completed smoothly but hoarseness was observed immediately after the operation. The cause was suspected to be the right recurrent laryngeal nerve palsy or manipulative injury due to intubation. No other complications were observed. The hoarseness persisted even after discharge. Thus, the patient visited a nearby otolaryngologist and was diagnosed with left vocal cord paralysis by laryngoscopy. Postoperative hoarseness has been present for more than 3 years, but no improvement has been seen. The patient was 157.0 cm tall and weighed 54.8 kg and used a 35 Fr double lumen tube for left intubation as the intubation tube. Pathological analysis revealed that it was a primary lung adenocarcinoma, pathological T1aN0.

### Case 2

**Fig. 2:**
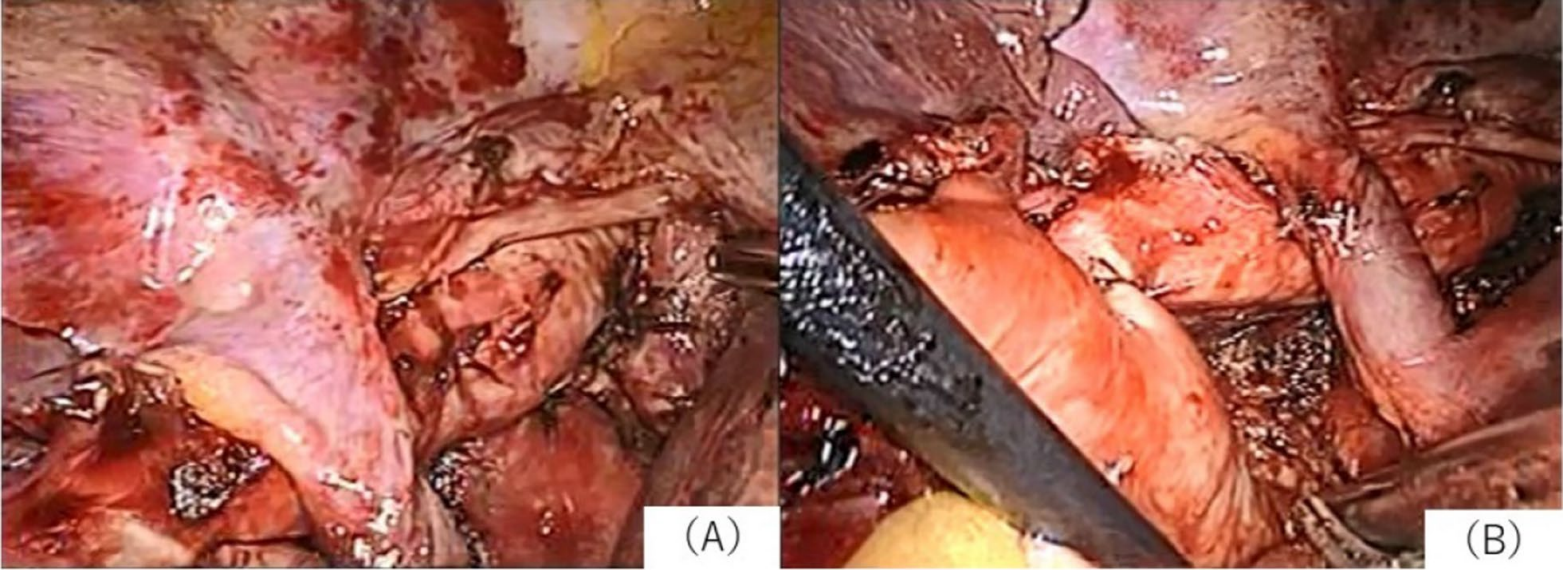
Case 2: this figure shows the findings after the upper longitudinal node dissection. **A** is after the dissection of # 2R, and **B** is after the dissection of # 4R

**Fig. 3:**
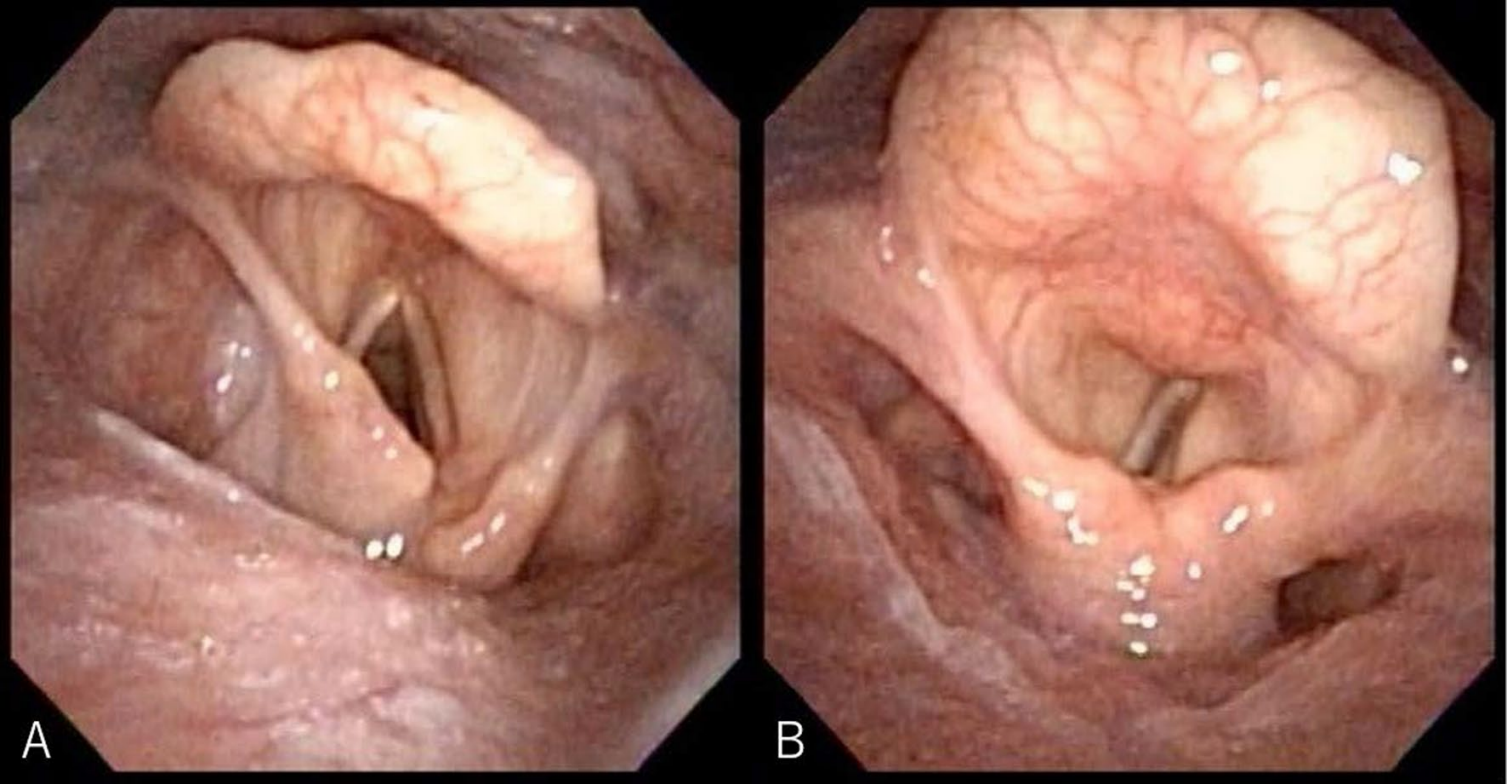
Case 2: this figure shows laryngoscopic findings during breathing (**A**) and phonation (**B**), showing that the left vocal cord is fixed

An 80-year-old woman was admitted to our hospital with suspected primary lung cancer in the right upper lobe clinical T1bN0M0, stage IA2. Thoracoscopic right upper lobectomy and superior mediastinal node dissection were performed under general anesthesia with selective lung ventilation using a double-lumen tube. Nodes in # 2R and # 4R were removed en bloc. Nodes in # 2R and # 4R were dissected along the anterior surface of the trachea (Fig. 2). The patient complained of hoarseness one day after the operation and consulted an otolaryngologist on the sixth day after the operation because no improvement was observed. Consequently, the patient was diagnosed with left vocal cord paralysis by laryngoscopy (Fig. 3). Postoperative hoarseness has been present for more than 3 years, but no improvement has been seen. The patient was 148.4 cm tall and weighed 46.8 kg and used a 32 Fr double lumen tube for left intubation as the intubation tube. Pathological analysis revealed that it was a primary lung adenocarcinoma, pT1aN0.

## Discussion

The standard treatment for lung cancer is lobectomy and lymphadenectomy. It has long been pointed out that superior mediastinal node dissection can cause ipsilateral recurrent laryngeal nerve paralysis. However, few reports of recurrent laryngeal nerve paralysis on the contralateral side of the surgical field are available [1].

The left recurrent laryngeal nerve branches off from the left vagus nerve. Eighty-five percent branch off 0.8–1.2 cm above the inferior margin of the aortic arch, passing just below the ductus arteriosus and recurring the aortic arch. It then ascends between the right side of the aortic arch and the anterior surface of the left bronchial root and gradually shifts to the central part of the left side of the trachea [2]. The anterior surface of the trachea is exposed and dissected in the right superior mediastinal node dissection. Thus, the vicinity of the left recurrent laryngeal nerve is operated upon. When dissecting lymph nodes # 2R and # 4R, we sometimes experience these nodes are interconnected with # 2L and # 4L nodes. In such cases, if the dissection is continued deeper into the left side, the dissection may reach the vicinity of the left recurrent laryngeal nerve.

Variations exist in the position of the recurrent laryngeal nerve branches from the vagus nerve [3]. In addition, reports of the abnormal running of the recurrent laryngeal nerve ascending around the pulmonary artery are available [4]. Thus, the recurrent laryngeal nerve could be displaced to the anterior or posterior surface of the tracheal wall. If the recurrent laryngeal nerve is displaced to the anterior surface of the trachea, the vicinity of the nerve is manipulated during contralateral node dissection, increasing the risk of recurrent laryngeal nerve injury.

In addition to the direct damage (e.g., recurrent laryngeal nerve transection), neuropathy may be caused by exclusion by operation in the vicinity or energization using an energy device in the vicinity.

Stimulation by intubation may be the cause of hoarseness rather than the operation of the surgical field. Arytenoid cartilage subluxation and recurrent laryngeal nerve injury are considered the causes of hoarseness due to intubation [5]. However, in this case, no evidence of arytenoid cartilage subluxation exists. Moreover, it is reported that excessive cuff pressure can cause recurrent laryngeal nerve damage [5]. However, in this case, cuff pressure was precisely controlled not to be excessive. In addition, in these cases, the intubation tube is sized to fit their physique.

Careful manipulation is required to prevent left recurrent laryngeal nerve injury during right superior mediastinal node dissection with contralateral recurrent laryngeal nerves in the vicinity if lymphadenopathy is significant.

The right lower paratracheal node dissection over the right lateral wall of the ascending aorta should be avoided when the lymphoadenopathy in the vicinity is not suspected. The right lateral wall of the ascending aorta is defined as the left side boundary plane for the # 4R station. Clearance beyond the right wall of the ascending aorta can cause contralateral recurrent laryngeal nerve damage and should be avoided.

## Conclusion

Node dissection is performed in the standard procedure for right upper lobe lung cancer. At this time, care must be taken not to cause damage not only to the recurrent laryngeal nerve on the ipsilateral side but also to the recurrent laryngeal nerve on the contralateral side.

## Data Availability

Data sharing is not applicable to this article as no data sets were generated or analyzed for the study.
